# Can mowing restore boreal rich-fen vegetation in the face of climate change?

**DOI:** 10.1371/journal.pone.0211272

**Published:** 2019-02-19

**Authors:** Louise C. Ross, James D. M. Speed, Dag-Inge Øien, Mateusz Grygoruk, Kristian Hassel, Anders Lyngstad, Asbjørn Moen

**Affiliations:** 1 Department of Natural History, NTNU University Museum, Norwegian University of Science and Technology, Trondheim, Norway; 2 School of Biological Sciences, University of Aberdeen, Aberdeen, United Kingdom; 3 Department of Hydraulic Engineering, Warsaw University of Life Science-SGGW, Warsaw, Poland; Ecole Pratique des Hautes Etudes, FRANCE

## Abstract

Low-frequency mowing has been proposed to be an effective strategy for the restoration and management of boreal fens after abandonment of traditional haymaking. This study investigates how mowing affects long-term vegetation change in both oceanic and continental boreal rich-fen vegetation. This will allow evaluation of the effectiveness of mowing as a management and restoration tool in this ecosystem in the face of climate change. At two nature reserves in Central Norway (Tågdalen, 63° 03’ N, 9° 05 E, oceanic climate and Sølendet, 62° 40’ N, 11° 50’ E, continental climate), we used permanent plot data from the two sites to compare plant species composition from the late 1960s to the early 1980s with that recorded in 2012–2015 in abandoned and mown fens. Changes in species composition and frequency were analysed by multivariate and univariate methods in relation to environmental variables and modelled climate and groundwater data. Mowing resulted in a decline in shrub and *Molinia caerulea* cover at the continental and oceanic sites respectively, and the total cover of specialist fen species had increased to a significantly greater extent in the mown plots than the unmown at the continental site. However, mowing did not have an effect on the cover of specialist bryophyte species, and some specialist species declined regardless of mowing treatment. Temperature sums had increased at both sites, but precipitation had not changed significantly. Mowing was shown to be the most important determinant of plant community composition at both sites, with local environmental conditions being of secondary importance. In conclusion, the abandonment of traditional management practices results in the loss of characteristic fen species. In order to encourage the restoration of typical rich-fen vegetation, particularly in oceanic areas, additional management measures, such as more intensive mowing, may be required.

## Introduction

Many ecosystems of high conservation value have been shaped by human activity, and rely heavily on the continuation of traditional management methods, particularly in grasslands and wetlands [1, 2]. Mowing is widely held to promote plant species diversity by increasing accessibility of light and heat [3, 4]. Fens are among the most diverse ecosystems in the boreal region [5], and are priority habitats for conservation that harbour a high number of specialised and endangered species [2, 6]. For centuries, fens were important for hay production and as pastures for livestock grazing throughout Europe. Regular harvesting turned large areas of fen vegetation into open semi-natural landscapes [7–11]. However, the widespread traditional use of fens ended many decades ago across most of Europe. The cessation of management often results in the displacement of specialist fen species following secondary succession of competitive species, particularly by graminoids and shrubs, and subsequent biomass accumulation [3, 6, 11, 12]. The grass *Molinia caerulea* is a particularly strong competitor in fens and can invade rapidly, causing a reduction in species richness [6]. Thus, traditionally-managed hay fens are threatened all over Europe, including Norway [13], where 105 of 246 Red-Listed vascular plant species (ca. 43%) are found primarily in semi-natural habitats [14]. Despite this, only 200 ha out of more than 300 000 ha of former hay fens are mown as a management measure [5]. There is, therefore, a clear need for restoration measures in this valuable habitat in order to increase the abundance of specialist fen species, both vascular plants and bryophytes, to maintain viable populations, and more research to facilitate evidence-based management for fen restoration.

Boreal fens require cool and humid climatic conditions [15], although the occurrence of fens is widely held to be driven primarily by local edaphic conditions, and to depend only secondarily on climate [5]. The effect of climate change, and its interaction with management, on boreal rich fen vegetation is currently unclear, although interactions between drivers of change can play an important role in determining plant communities [16]. Plant species in this region are often particularly sensitive to climatic warming [17, 18], and fen vegetation may be vulnerable to shifting hydrological fluxes [19] and the predicted changes in precipitation [15]. The effect of climate on fen species can also vary along an oceanic—continental gradient, i.e. between coastal and inland areas [18, 20].

Detailed botanical and environmental monitoring over a period of many decades provides a rare opportunity to assess vegetation change driven by land management and climate change over an ecologically-significant timescale, and to integrate climate change into management strategies aimed at conserving biodiversity [21–24]. In this study, we used data from a historical dataset on boreal fens based on permanent plots established in the late 1960s and early 1970s at an oceanic and a continental site in Central Norway [25], to assess long-term change in parallel communities of abandoned and harvested fens (overgrown for >60 years and mown for 30 years respectively). We are thus able to address the long-term effect of mowing under different climatic regimes. We used a re-survey approach to (1) assess how fen vegetation has changed over this period, both with and without mowing, in terms of species composition and diversity, (2) examine how the impact of mowing varies with climatic and environmental differences between oceanic and continental areas, and (3) provide evidence as to whether mowing is an effective restoration tool for boreal fens.

## Materials and methods

### Study areas

**Fig 1:**
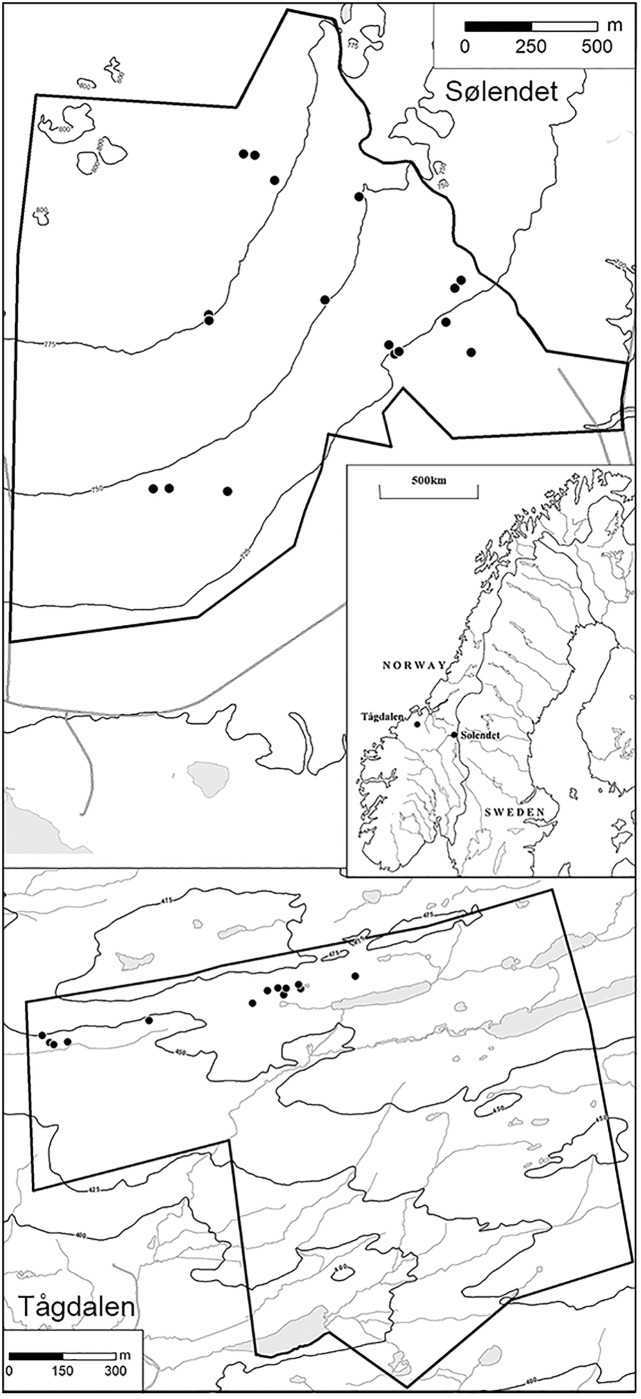
Map of locations of study site and localities. The location of Sølendet (continental site; 62° 40’ N, 11° 50’ E) and Tågdalen (oceanic site; 63° 03’ N, 9° 05’ E) nature reserves in Central Norway and the positions of localities within. The shortest distance between localities is more than 10 m.

The study was carried out in two nature reserves 145 km apart in Central Norway (Fig 1), the coastal Tågdalen reserve in Surnadal and the inland Sølendet reserve in Røros (S1 Table). Both areas lie at the transition between the middle and northern boreal vegetation zones (zonation according to [25, 26]), and are characterized by a short growing season lasting from late May to late August. The areas are dominated by base-rich bedrocks such as grey-green phyllite, mica schist and greenstone [27]. The bedrocks and the moraine yield a base-rich, fine-grained soil that is readily waterlogged, leading to paludification (peat formation). The study areas are dominated by sloping fens (slope > 3°; generally steeper at the oceanic site (up to >20°) than at the continental site) and birch woodland, with rich fens covering large areas [7, 11]. Rich fens are peat-forming and with characteristic vegetation dominated by brown mosses (species in Amblystegiaceae and Calliergonaceae), reflecting a supply of base-rich water (pH most often > 6). Poor fens have a lower pH and are dominated by bog mosses (*Sphagnum* species) in the ground layer. The fens analysed consist of carpet and lawn communities of fen expanses, and fen margin communities [11, 28]. At both sites, the vegetation is dominated by *Trichophorum cespitosum*, *M*. *caerulea*, several *Carex* species, and *Campylium stellatum*. *Scorpidium cossonii* and *Thalictrum alpinum* are also very common. The continental site includes some alpine/continental species, such as *Equisetum variegatum* and *Pedicularis oederi*, whereas lowland/oceanic species such as *Schoenus ferrugineus* and *Narthecium ossifragum* characterise the oceanic site. These rich fens were used for haymaking for centuries until traditional mowing ceased around 1950. Although only one site of each type (continental and oceanic) were compared, these sites are nevertheless representative of fens in those climatic regions, and represent important case study areas for this vegetation type. In the phytosociological classification system of fen vegetation [29], the vegetation plots mainly belong to the alliances Caricion atrofusco-saxatilis (upper boreal/alpine/arctic rich fens; Sølendet) and Caricion davallianae (mainly nemoral-lower boreal rich fens; Tågdalen). A few plots may be classified to the alliances Sphagno-Tomentypnion (Sphagnum-brown-moss dominated rich fen) and Stygio-Caricion limosae (boreal carpet rich fen). Further descriptions of the study areas, vegetation types and environmental data is found in [7, 11, 25].

### Botanical data

The localities at both sites (17 from the continental site, 13 from the oceanic) were established between 1967 and 1983 in selected areas of homogeneous vegetation, where permanent plots of 25m^2^ were marked and initial phytosociological analyses made. Half of the plot (or an adjacent plot) in each locality has then been mown regularly every second year in the first half of August, resembling the traditional use of these areas [11, 30]. The other 12.5m^2^ was set aside as a reference plot and left unmown. The plots were re-surveyed between 2012 and 2015. In each locality, therefore, phytosociological data exist for the following three types of plots: “initial”, analysed >40 years ago, after being unmown for >20 years (25 m^2^); “re-survey unmown”, unmown for >60 years (12.5 m^2^); “resurvey mown”, plots that, after being unmown for >20 years, have been mown every second year for > 30 years (12.5 m^2^). Based on studies of the species-area curve in these vegetation, the 25m^2^ plots include 6% more species than the 12.5m^2^ plots (Øien & Moen, unpublished data). This gives rise to the four site types into which plots are classified and which we used in analysis, where the first letter refers to the site (continental (C) or oceanic (O), the second to the change between the initial survey and either the unmown (u) or mown (m) plots of the re-survey, i.e. Cu, Cm, Ou and Om.

We sampled plots in homogenous stands of vegetation and estimated species cover on a six-degree scale. In each plot, the cover of each species was estimated using a scale close to the Hult-Sernander-DuRietz scale [31], with the classes 1: < 6.25%, 2: 6.3–12.5%, 3: 12.6–25%, 4: 26–50%, 5: 51–75% and 6: 76–100%. In some plots, we estimated species cover at a finer resolution, but all analyses were transformed to the referred scale. All cover class values were then converted to percentage cover using the mid value of each cover class as percentage cover before data analysis [7]. All phytosociological sampling made by AM or by AM and D-IØ together, with some assistance from KH on the bryophytes. Both the homogeneity and the cover of species were judged subjectively, bringing some inaccuracy into the analyses, as can also occur through differences between plots inside the localities [32]. Results from the data analyses were therefore interpreted with caution. Nomenclature follows Elven [33] for vascular plants and Frisvoll et al. [34] for bryophytes.

### Environmental data

The Norwegian Meteorological Institute provided spatially interpolated estimates of temperature and precipitation for both sites from 1960 onwards, with target elevations of 460 and 725 m above sea level (a.s.l.) for Tågdalen and Sølendet respectively. Temperature estimations were modelled with residual kriging [35], and data from a weather station that were established at each site in 2007 (a few hundred meters from the localities) were included in the estimates. Effective temperature sum (ETS) were calculated using a formula developed for the boreal zone [36]: $ETS=\sum_{n=a}^{b}Tm-5{}^{\circ}$, where T_*m*_ is mean daily temperature, and a and b are the third day of the first and last snowfree, five-day period with mean T_*m*_ > 5 °C respectively [18]. Precipitation sums were the annual volume of rainfall at each site (mm). Mean peat depth and pH in water in each locality was measured in each plot. At Tågdalen this was done when the localities were established (1967–69), and at Sølendet this was done in 1981. All measurements was carried out in August or September. Production is the dry aboveground biomass measured when the localities were first mown after > 20 years of abandonment. Groundwater levels within both sites were modelled using the MODFLOW-based approach [37]. Using this approach we developed transient groundwater flow models in order to simulate the response of the fen’s hydrology to boundary conditions defined by groundwater recharge, evapotranspiration and natural drainage. As the geology at both sites is similar, developed models simulated groundwater flow in peat and underlying strata of morainic sediments. Models were calibrated using groundwater level data collected in Tågdalen and Sølendet in 2009–2013. Simulations of groundwater level fluctuations in each of the sites analysed were based on 10-day intervals for the period 2001–2013. Hydrological conditions of the sites were described by mean, median, maximum and minimum groundwater depths (m), magnitude (range between the highest and the lowest water levels modelled, m), duration (time, which particular groundwater depths lasted for, days) and inundation (days/year). A summary of the environmental data are given in S3 Table.

### Data analysis

The data were analysed in the R statistical environment (R Development Core Team 2015, version 3.2.3) running the package *vegan* (Oksanen et al. 2014, version 2.4–0); the functions used are shown in italics.

The change in the cover of shrubs, *M*. *caerulea*, specialist vascular plant species and specialist bryophytes was calculated for unmown and mown plots at each site. See Table 1 (taken from [7]) for species designated as being moderately or extremely rich fen specialists. To estimate the effect of different plot size on the number of species per plot, the number of species were counted in plots of increasing size within a few of the localities, and species-area curves were drawn so that the appropriate values could be interpolated (Øien & Moen, unpublished data). These change metrics were then used as response variables in a linear mixed effects model (*lme*), with mowing treatment and the interaction between mowing treatment and time between surveys (years) as fixed effects, and locality as a random effect, as the mown and unmown pairs are not independent of each other. The interaction between mowing treatment and time between surveys was included as there were differences in the length of the intersurvey period between plots. Further, we proceeded with one-way ANOVA (*aov*) and Tukey *post hoc* tests (*TukeyHSD*) comparing the change metrics between the unmown and mown plots for each site type, i.e. two sites and two treatments.

**Table 1:** Specialist plant species of moderately or extremely rich fens. Species designated as moderately or extremely rich fen specialists for vascular plants and bryophytes, i.e. those that commonly occur in moderately or extremely rich boreal mire vegetation of Central Norway (taken from [7], p. 197).

Vascular plants	Bryophytes
*Salix myrsinifolia*	*Bryum pseudotriquetrum*
*Bartsia alpina*	*Calliergon richardsonii*
*Crepis paludosa*	*Calliergonella cuspidata*
*Dactylorhiza incarnata* ssp. *cruenta*	*Campylium stellatum*
*Dactylorhiza incarnata* ssp. *incarnata*	*Catoscopium nigritum*
*Gymnadenia conopsea*	*Cinclidium stygium*
*Listera ovata*	*Ctenidium molluscum*
*Parnassia palustris*	*Fissidens adianthoides*
*Pedicularis oederi*	*Fissidens osmundoides*
*Saussurea alpina*	*Plagiomnium elatum*
*Saxifraga aizoides*	*Plagiomnium ellipticum*
*Thalictrum alpinum*	*Rhizomnium pseudopunctatum*
*Tofieldia pusilla*	*Scorpidium scorpioides*
*Triglochin palustris*	*Tomentypnum nitens*
*Carex atrofusca*	*Leiocolea rutheana*
*Carex buxbaumii*	
*Carex capillaris*	
*Carex capitata*	
*Carex flava*	
*Carex hostiana*	
*Carex microglochin*	
*Carex pulicaris*	
*Eleocharis quinqueflora*	
*Eriophorum latifolium*	
*Juncus castaneus*	
*Juncus triglumis*	
*Kobresia simpliciuscula*	
*Schoenus ferrugineus*	

Multivariate analyses were performed using non-linear multi-dimensional scaling (NMDS) (*metaMDS*) on the species data, which were square-root transformed and the analysis used the Bray-Curtis dissimilarity measure. NMDS was selected as the preferred type of analysis as it has been shown to be robust for nonlinear relationships of species abundances along long ecological gradients, accurately representing underlying dissimilarities [38]. The environmental variables (slope angle (°), mean peat depth (cm), pH of water, electrical conductivity, (μS/cm), above-ground production (g/m^2^), and the groundwater variables detailed above) were fitted on to the ordination using *envfit*. An ordination diagram was plotted showing the unmown and mown plots from both sites, the fen specialist and species indicative of succession and fen discontinuity (*M*. *caerulea* and *Betula* species), and the significant (*P*<0.01) environmental variables (mean peat depth, slope angle, pH of water, median groundwater level and maximum groundwater level). As some of the groundwater variables were collinear, only those that were significant and had the highest R^2^ values were included. Linear regression was used to test the significance of the changes in temperature (°C) and precipitation sums (mm) over time (*lm*). The cover of fen specialist species (S1 Table), plus the succession-indicating species *M*. *caerulea*, *Betula nana* and *Betula pubescens*, were tested for significant differences between the initial and unmown and initial and mown plots using paired t-tests (*t*.*test*). In view of the large number of tests, a more rigorous significance cut-off was applied when testing for differences in species cover, with only *P*<0.01 being considered significant, and the species data were log-transformed using the common logarithm (log_10_) prior to analysis.

## Results

**Fig 2:**
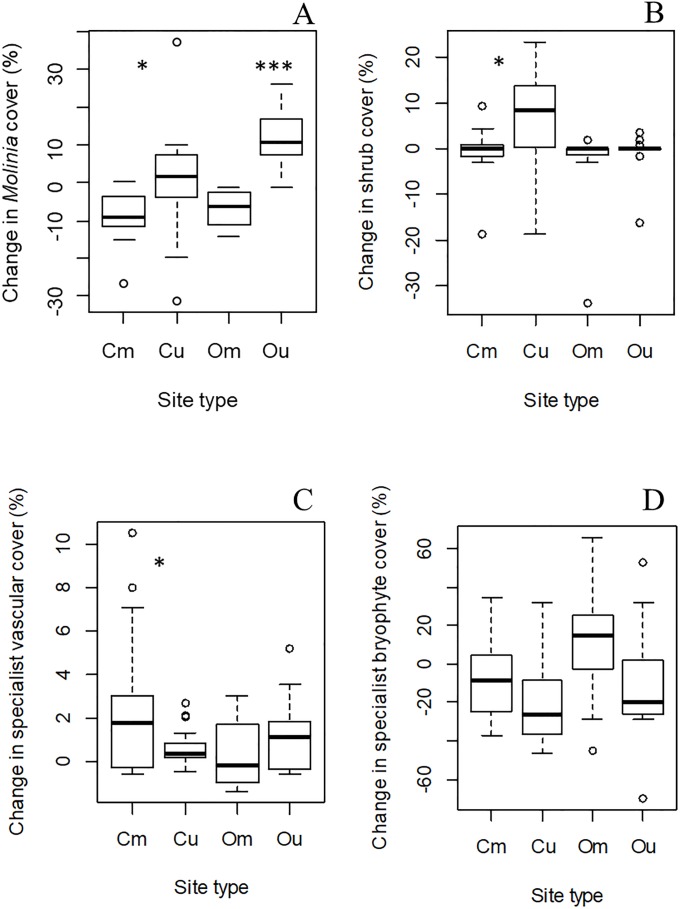
Boxplots of change metrics. Change in *M*. *caerulea* cover (%) (A), change in shrub cover (%) (B), change in specialist vascular plant cover (%) (C) and change in specialist bryophyte cover (%) (D). Site types: Cm = continental mown (*n* = 17); Cu = continental unmown (*n* = 17); Om = oceanic mown (*n* = 13); Ou = oceanic unmown (*n* = 13). ***: P ≤ 0.001; **: P ≤ 0.01; *: P ≤ 0.05 for significant differences between means of unmown and mown plots at each site in Tukey *post hoc* tests.

The results of the linear mixed models show that specialist bryophyte species cover is not affected by mowing (Table 2). However, mowing resulted in a decline in shrub cover, and an increase in specialist fen vascular plant species cover at the continental site, and a decline in *M*. *caerulea* cover at both sites, particularly the oceanic site (see also Fig 2). There was also a significant interaction between mowing treatment and time for the change in *M*.*caerulea* cover.

**Table 2:** Results of linear mixed models (with locality as a random factor) of vegetation change metrics versus mowing treatment and the interaction between mowing treatment and time.

Change metric	Mowing treatment	Mowing treatment*Time
Shrub cover	-3.52**	n.s.
*M*. *caerulea* cover	-6.76***	3.81***
Specialist fen vascular plant cover	2.20*	n.s.
Specialist fen bryophyte cover	n.s.	n.s.

Vegetation metrics refer to change in the metric between surveys. Explanatory variables refer to the mowing treatment (unmown or mown), and the interaction between mowing treatment and time (years) between surveys. T values are shown for each variable in each model.

Significance of the variables is shown by asterisks:

* = P<0.05;

** = P<0.01;

*** = P<0.001.

Df = 59 for all models.

Graphs of variables are shown in Fig 2.

At both sites, particularly the oceanic site, mowing has resulted in a decline in *M*. *caerulea* cover, which increased in the absence of mowing at the continental site (C_unmown_ = 11.5% to 12.5%; *P* = 0.434, *n* = 17; C_mown_ = 11.5% to 4.1%, *P* = 0.001, *n* = 17; O_unmown_ = 10.1% to 22.3%, *P*<0.001, *n* = 13; O_mown_ = 10.1% to 3.4%, *P*<0.001, *n* = 13; see also Fig 2). Shrub cover remained fairly static at both sites and mowing treatments, although there was more variability in the unmown plots of the continental site (C_unmown_ = 6.0% to 10.2%; *P* = 0.114, *n* = 17; C_mown_ = 6.0% to 1.0%, *P* = 0.123, *n* = 17; O_unmown_ = 2.5% to 1.8%, *P* = 0.587, *n* = 13; O_mown_ = 2.5% to 0.5%, *P* = 0.365, *n* = 13; see also Fig 2). This was largely due to the low cover of shrubs at the oceanic site, whereas *B*. *nana* invades many unmown plots at the continental site. Specialist vascular plant cover increased with mowing at the continental site (3.2% to 5.6%, *P*<0.001, *n* = 17), whereas specialist bryophytes declined in the absence of mowing at the same site (60.3% to 39.7%, *P*<0.001, *n* = 17), although there was no such difference at the oceanic site.

**Fig 3:**
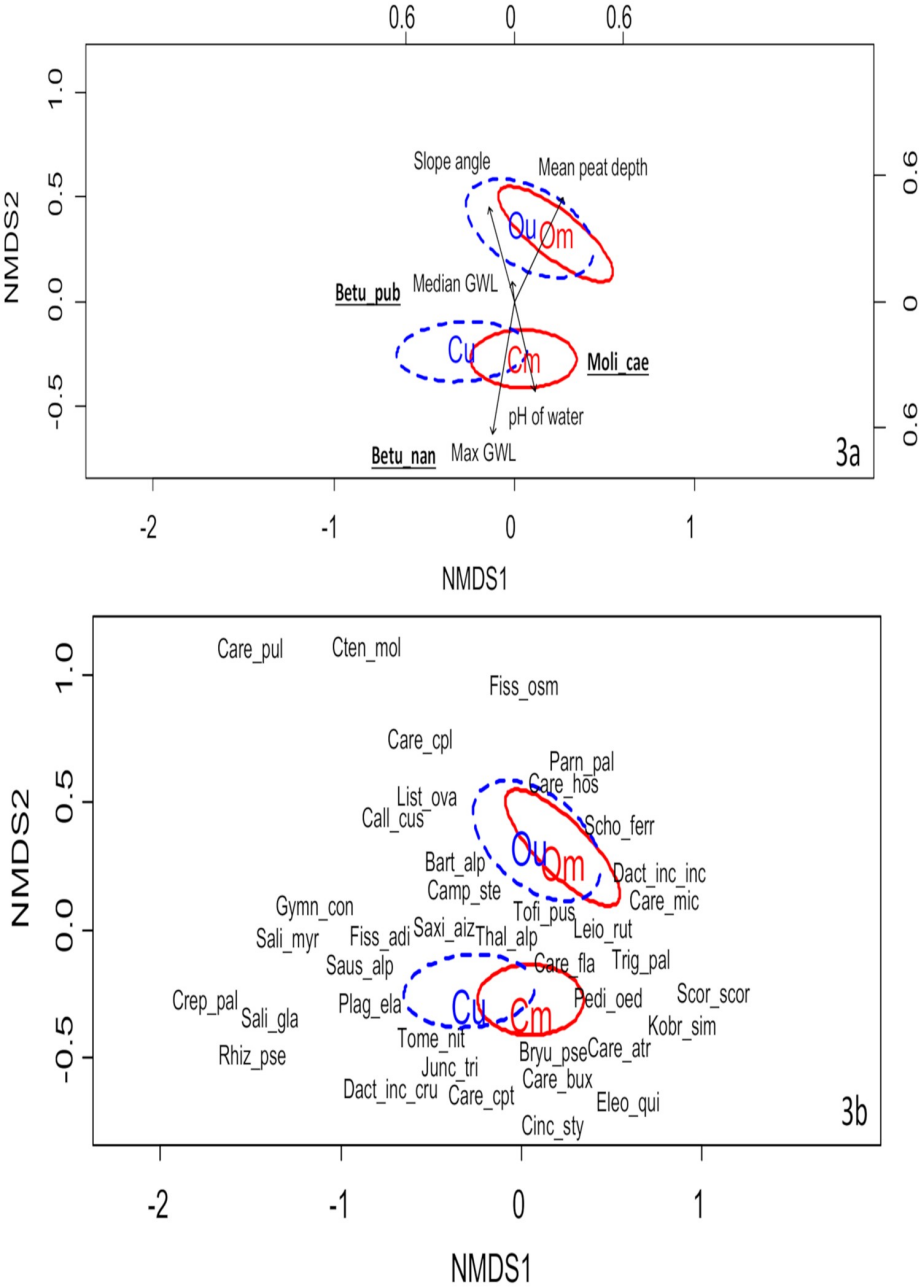
Ordination diagram of non-linear multidimensional scaling (NMDS) analysis. Species data were square-root transformed, and the analysis used the Bray-Curtis dissimilarity measure. 3a shows plots from both sites and mowing treatments. Solid red lines show standard error ellipses (confidence limit = 0.95) calculated from the sample scores for the mown plots; dashed blue lines for the unmown plots. Position of centroids are shown by ellipse labels. Succession indicating species (*M*. *caerulea*, *B*. *nana* and *B*. *pubescens*) are shown in bold. Black arrows show length and direction of significant environmental variables (*P* < 0.01) where the labels are underlined: mean peat depth (cm), slope angle (°), pH of water, median groundwater level (GWL) (m) and maximum groundwater level (m). Site types: Cm = continental mown (*n* = 17); Cu = continental unmown (*n* = 17); Om = oceanic mown (*n* = 13); Ou = oceanic unmown (*n* = 13). 3b shows plots from both sites and mowing treatments, and specialist fen species (see Table 1 and S2 Table). See S2 Table for species abbreviations and summary data on changes in cover of individual species.

The NMDS used the Bray-Curtis dissimilarity index to analyse two dimensions with accepted stress of 0.153392. Mowing has resulted in a shift in species composition at both sites, although there is still a considerable degree of overlap, particularly at the oceanic site, where the mown plots are grouped in a smaller ellipse on the ordination diagram, and are therefore noticeably less variable than the unmown (Fig 3). Although both sites were analysed in a single NMDS, different variables were associated with each site. At the continental site, the environmental variables with the best fit in the model are pH of water (R^2^ = 0.191, *p* = 0.007, *n* = 16) and maximum groundwater level (R^2^ = 0.409, *p* = 0.001, *n* = 16), while at the oceanic site, these variables are slope angle (R^2^ = 0.224, *p* = 0.004, *n* = 10), peat depth (R^2^ = 0.318, *p* = 0.001, *n* = 10) and median groundwater level (R^2^ = 0.529, *p* = 0.001, *n* = 10). Many of the specialist fen species are strongly associated with the mown plots, although *Carex pulicaris*, *Salix myrsinifolia* and *Crepis paludosa* are notable exceptions. Relatively few fen specialists are associated with the oceanic site, although *P*. *palustris*, *S*. *ferrugineus*, *Carex microglochin* and *Dactylorhiza incarnata* spp. *incarnata* characterise the mown plots there. Unsurprisingly, *M*. *caerulea* and *B*. *pubescens* are strongly associated with the unmown plots of the oceanic site. At the continental site, the unmown plots are characterised by *B*. *nana* and *Saussurea alpina*, and the mown plots by, amongst others, *Kobresia simpliciuscula*, *Bryum pseudotriquetrum*, *Carex atrofusca* and *P*. *oederi* (Fig 3).

S2 Table shows summary data on mean cover changes in each fen specialist and succession indicating species. Of those at the continental site, *T*. *alpinum*, *C*. *stellatum* and *Carex capillaris* declined in cover in the unmown plots; only *Cinclidium stygium* increased significantly here. In the continental mown plots, *M*. *caerulea* and *Fissidens adianthoides* declined, while only *Carex flava* increased. At the oceanic site, *M*. *caerulea* increased substantially in the unmown plots and declined in the mown. The cover of *P*. *palustris* and *T*. *pusilla* declined in both mown and unmown plots. *C*. *capillaris* declined in the unmown plots, and *S*. *alpina* and *B*. *pseudotriquetrum* declined in the mown plots at the oceanic site.

**Fig 4:**
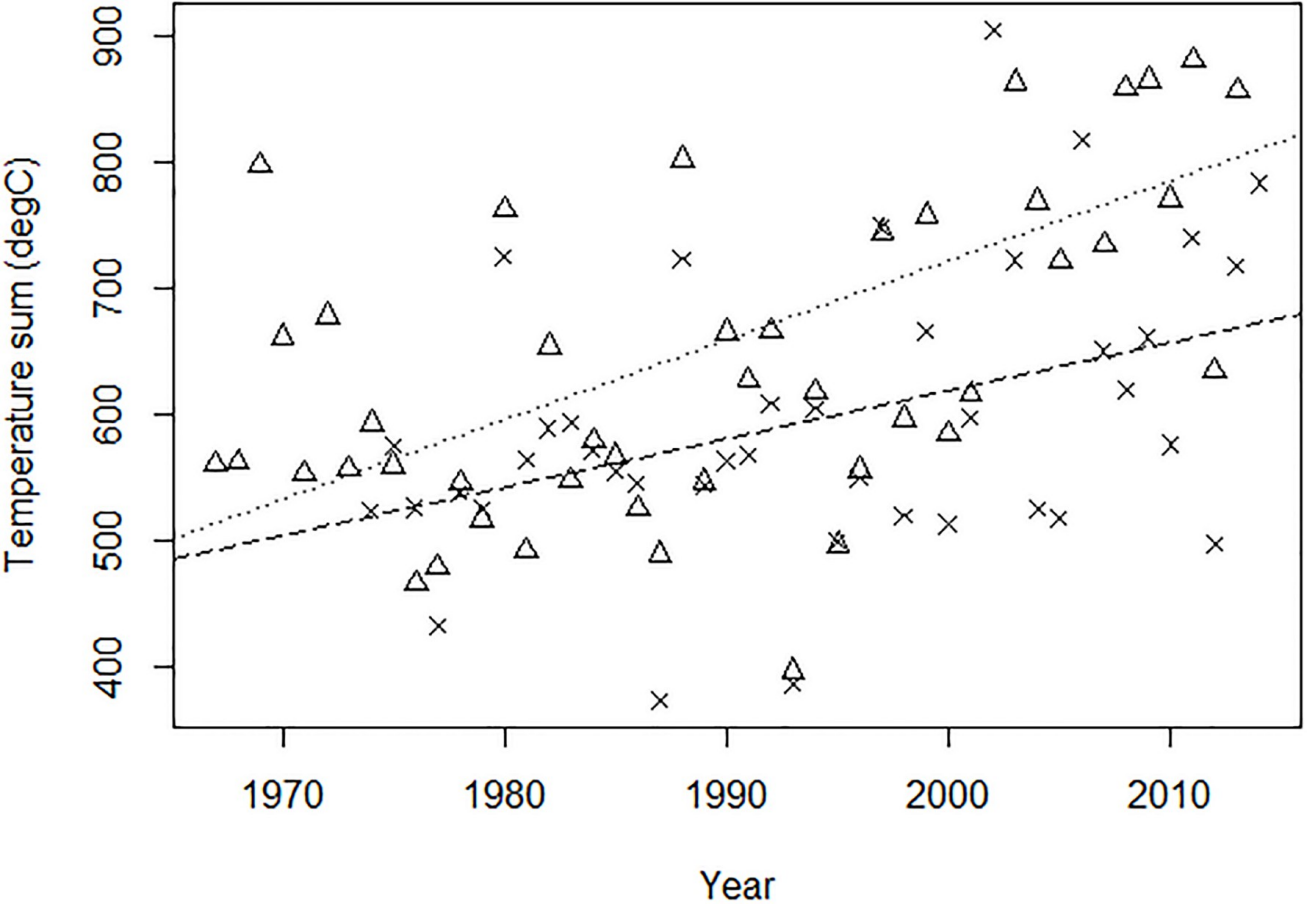
Temperature sums (°C) for the continental (1974–2014; crosses) and oceanic (1967–2014; triangles) sites. Points show temperature sums measured each year at both sites. Regression lines are plotted from linear models of the continental (dashed line: r^2^ = 0.149, *P* = 0.007, *n* = 41) and oceanic (dotted line: r^2^ = 0.340, *P*<0.001, *n* = 48) site data.

Linear models showed that the temperature sums have increased significantly between 1967/74 and 2014 (Fig 4: oceanic: R^2^ = 0.340, *p*<0.001; continental: R^2^ = 0.149, *p* = 0.007), but the precipitation sums have not changed significantly (oceanic: R^2^ = 0.001, *p* = 0.309; continental: R^2^ = 0.032, *p* = 0.137). At the oceanic site, the mean temperature and precipitation sums are significantly higher than at the continental site (temperature: +64.7°C, *P* = 0.021; precipitation: +922.7mm, *P*<0.001).

## Discussion

This study used historical botanical and environmental data to highlight the importance of land management in restoring boreal rich-fen vegetation in a changing climate. The changes detected here can therefore be interpreted with confidence, in contrast to multiple short-term studies that may fail to detect important trends [39]. Our results suggest that the effect of mowing is an important factor in determining vegetation composition at these rich fens in Central Norway. In the period between the initial survey and re-survey of these two rich fen study areas, concurrent climate change has occurred, with significant increases in temperature sums. Local environmental conditions differ markedly between regions, but play a secondary role. Vegetation change since the beginning of the experiment appears to be greater at the continental site than the oceanic, and in the mown than the unmown plots. However, although mowing prevents the encroachment of *M*. *caerulea* and *Betula* species, this practice may not be sufficient to over-ride the effect of other environmental drivers, including climate change, on all fen specialist species.

We have revealed that different vegetation change metrics—change in the cover of *M*. *caerulea*, shrubs, specialist vascular plants and specialist bryophytes—respond to mowing in different ways. At both sites we observed a decrease in *M*. *caerulea* cover in the mown plots, although there was also a significant interaction with time between surveys for this metric, implying that the changes could be a result of differences in the timing of the initial set up of the plots (up to 17 years). However, this is likely to be related to the large increase in *M*. *caerulea* in the unmown plots at the oceanic site. Shrub cover also declined at the continental site. The suppression of *M*.*caerulea* and shrubs by mowing is likely to be the most important factor in maintaining typical open fen vegetation [26]. Mowing has been linked with the persistence and restoration of fens by preventing litter accumulation and reducing the density of the field layer, thus reducing competitive interactions and creating space for less competitive, light-demanding species [3, 6, 10, 40, 41]. Similar outcomes have been recorded in other anthropogenic vegetation types, including prairie grassland [42], temperate grassland [43, 44], wet meadows [45] and calcareous sand dune meadows [4]. Mowing has also been found to favour certain rare species such as the orchid *Dactylorhiza lapponica* [46] and the grassland endemic *Gentianella praecox* ssp. *bohemica* [47]. However, in our study, the decline of species such as *P*. *palustris* and *T*. *pusilla* occurred regardless of mowing, showing that other environmental drivers are also affecting the vegetation. Although grazing would appear to provide a similar type of disturbance to mowing, the introduction of grazing to a fen may cause extensive damage from trampling due to the wet conditions, creating a more heterogeneous habitat as a result [26]. In a few examples were grazing was used as a restoration measure in a fen or fen meadow, the cover of fen specialists in grazed areas did not increase relative to similar ungrazed areas [48], or contained fewer fen specialist species than mown areas [40]. Mowing therefore appears to be particularly effective in promoting specialist vascular plant species, which suggests that the propagules of these species are still present, strengthening the argument for restoration before abandonment causes these species to disappear. However, the same is not the case for bryophytes, which could indicate a response to climate warming. Differences in response to mowing between the different species groups occur because of differences in morphology, physiology and ecology [6]. Bryophytes lack roots for active water uptake from the ground, and are poikilohydric, whereby water and nutrients are absorbed over the whole surface of the plant [49]. Consequently, bryophyte growth may be more dependent of precipitation patterns than groundwater levels. Bryophytes may therefore respond more negatively to climate change than vascular plants, and may also indicate such changes earlier [2].

From a conservation point of view, it is concerning if the management of fens does not results in the restoration that was targetted. A decline in bryophyte diversity and cover as a consequence of fen abandonment has been well-documented [9, 41, 50], and a positive influence of mowing on the coverage of endangered bryophyte species has also been reported [51]. A reversal in the decline of bryophyte cover will depend on disturbance (e.g. mowing and trampling), their ability to compete for space, and their capability for generative reproduction, which varies considerably between species [6]. The continental site has a higher water pH value and is more productive than the oceanic site, probably because of a stronger influence of oxygen-rich spring water. A more productive fen will have less space and be drier and shadier at ground level, limiting fen-adapted species. The increase in field layer productivity may have been exacerbated by climate warming, further increasing the competition for light and space. Factors that counteract this process through breaking up the field layer and creating space for less competetive bryophytes are trampling by large herbivores and rodent winter activity [52]. There were fewer significant changes in the vegetation at the oceanic site compared to the continental. It may be that this is a result of the buffering effect of the high precipitation and humidity that characterise an oceanic climate [53]. However, the sample size at the continental site is higher (*n* = 17) than at the oceanic site (*n* = 13), which may have influenced the occurrence of significant results in the statistical tests.

Although we found that mowing was an important determinant of fen vegetation, climate change is also likely to have been important. The increase in temperature between the surveys is likely to have increased fen productivity at both sites, presenting a further threat to habitat specialists, as the lowering of soil moisture is disastrous for fen vegetation [54]. The temperature of fen soils is generally less variable and less extreme than air temperatures, suggesting a buffer effect of groundwater systems that may render them relatively resilient to climate change [55]. This explains the wide distribution range of many fen species in Europe [56]. Indeed, the cool waters from underground aquifers that saturate fens are expected to keep temperatures lower during warm periods and vice versa [55]. However, the buffer effect is likely to be reduced in warmer temperatures, when the soils are relatively sensitive to air temperature through being less water-saturated because of warmer air and solar radiation [55]. Fens may also be extremely sensitive to the predicted changes in precipitation [15], which could further compromise the buffer effect. An oceanic climate is characterised by high precipitation and humidity, with a higher frequency of waterlogging than continental areas [53]. However, because of the higher paludification rate caused by the humid conditions in oceanic regions, fens in such regions often include steep sloping fens which have drier peat than flatter fens in continental areas [5]. Despite these factors, mowing can have an even greater effect; land management was also shown to be more important than climate change in its effects on grassland functioning [16].

Future community composition of rich fen vegetation is likely to depend both on local management and on climate change. Without mowing, shrubs and graminoids, particularly *M*. *caerulea*, are increasingly likely to out-compete the specialist fen species that we are aiming to restore, particularly at oceanic sites where higher productivity induced by warmer temperatures could result in increasingly favourable conditions for *M*. *caerulea* growth. Although mowing re-sets the fen system to some extent, this may not be sufficient to ensure the persistence of specialist bryophyte species, especially in the face of climate change. Despite mowing not being effective in restoring all fen specialist species, particularly bryophytes, the potential of this type of management to mitigate against climate change impacts on fen vegetation, by removing increased plant biomass generated by extended growing seasons to create the more open conditions required by fen specialists, is considerable. Traditional management by mowing at least every second year should be continued, as abandoned fens lose habitat specialists and can lead to *M*. *caerulea* encroachment, particularly in the margins and in oceanic areas. More intensive management measures such as annual mowing for shorter or longer periods may be required to restore fully the cover of fen bryophyte species. Mowing practices could be scaled up to be carried out in similar fens where abandonment has led to the loss of typical fen species. Hydrological “buffer zones” around fens should be implemented to protect the hydrology of the sites in view of continuing temperature increases. Finally, species and communities that are broadly distributed geographically are likely to vary in their responses to change due to regional and local differences in habitat heterogeneity; these differences must be accounted for when addressing the dual effects of land management and climate change.

## Supporting information

S1 Table Characteristics of the study sites [20, 25]. Tågdalen climate data 1973–2008, Sølendet climate data 1974–2008.
(PDF)

S2 Table Percentage cover of fen specialist species and succession indicating species at both sites from both surveys. Initial survey (1960s-1980s; ci = continental initial, oi = oceanic initial); unmown plots (2012–2015; cu = continental unmown, om = oceanic unmown); mown plots (2012–2015; cm = continental mown, om = oceanic mown). Br = bryophyte, Fo = forb, Gr = graminoid, Sh = shrub. Cover changes in bold are significant (*P* < 0.01). *T* refers to the *t*-statistic from paired *t*-tests. Species with an asterisk (*) are classed as indicative of succession in rich fens.
(PDF)

S3 Table Environmental variables at the continental and oceanic sites. The “Difference” column shows difference in mean value between the continental and oceanic sites for that variable. *P* values that show significant differences between sites (*P*<0.05) are shown in bold.
(PDF)
