# Supplementary material for: Can mowing restore boreal rich-fen vegetation in the face of climate change?
Source: PLoS One. 2019 Feb 19;14(2):e0211272. doi: 10.1371/journal.pone.0211272 (PMC6380559; doi:10.1371/journal.pone.0211272)
Supplement: S1 Table — Tågdalen climate data 1973–2008, Sølendet climate data 1974–2008. (PDF) [file pone.0211272.s001.pdf]

Table S1. Characteristics of the study sites [20, 25].

| | **Sølendet (continental)** | **Tågdalen (oceanic)** |
|---|---|---|
| Latitude, longitude | 62° 40' N, 11° 50' E | 63° 03' N, 9° 05' E |
| Altitude (m a.s.l.) | 700-800 | 380-490 |
| Area (ha) | 306 | 146 |
| Mean annual precipitation (mm) | 637 | 1583 |
| Min. and max. annual precipitation (mm) | 471-862 | 968-2137 |
| Mean January temperature (°C) | -9.5 | -2.7 |
| Mean July temperature (°C) | 10.5 | 11.2 |
| Fen community type | *Caricion atrofusco-saxatilis* | *Caricion davallianae* |
| Number of localities | 17 | 13 |

Tågdalen climate data 1973-2008, Sølendet climate data 1974-2008.
