# Supplementary material for: Can mowing restore boreal rich-fen vegetation in the face of climate change?
Source: PLoS One. 2019 Feb 19;14(2):e0211272. doi: 10.1371/journal.pone.0211272 (PMC6380559; doi:10.1371/journal.pone.0211272)
Supplement: S2 Table — Initial survey (1960s-1980s; ci = continental initial, oi = oceanic initial); unmown plots (2012–2015; cu = continental unmown, om = oceanic unmown); mown plots (2012–2015; cm = continental mown, om = oceanic mown). Br = bryophyte, Fo = forb, Gr = graminoid, Sh = shrub. Cover changes in bold are significant (P < 0.01). T refers to the t-statistic from paired t-tests. Species with an asterisk (*) are classed as indicative of succession in rich fens. (PDF) [file pone.0211272.s002.pdf]

Table S2: Percentage cover of fen specialist species and succession indicating species at both sites from the initial survey (1960s-1980s; ci = continental initial, oi = oceanic initial) to the unmown plots (2012-2015; cu = continental unmown, om = oceanic unmown) and the mown plots (2012-2015; cm = continental mown, om = oceanic mown). Br = bryophyte, Fo = forb, Gr = graminoid, Sh = shrub. Cover changes in bold are significant ($P < 0.01$). *T* refers to the *t*-statistic from paired *t*-tests. Species with an asterisk (*) are classed as indicative of succession in rich fens.

			Continental site							Oceanic site						
Species	abbreviation (see Fig. 3)	plant group	Initial % cover	Unmown % cover	Δ in unmown	Mown % cover	Δ in mown	*T* Δ Unmown (df=16)	*T* Δ mown (df=16)	Initial % cover	Unmown % cover	Δ in unmown	Mown % cover	Δ in mown	*T* Δ Unmown (df=16)	*T* Δ mown (df=16)
Bryum pseudotriquetrum	Bryu_pse	Br	2.4	3.6	+1.2	4.4	+2.0	-0.74	-1.10	2.1	1.7	-0.4	0.5	**-1.6**	1.09	2.24
Calliergon richardsonii	Call_ric	Br	0.1	0.0	-0.1	0.0	-0.1	1.00	-1.0	0.0	0.0	0.0	0.0	0.0	n/a	n/a
Calliergonella cuspidate	Call_cus	Br	0.0	0.0	0.0	0.0	0.0	n/a	n/a	5.3	14.1	+8.8	0.0	-5.3	-1.65	1.14
Campylium stellatum	Camp_ste	Br	47.6	25.9	**-21.7**	43.0	-4.6	4.52	1.25	36.6	31.4	-5.2	49.2	+12.6	0.94	-1.32
Catoscopium nigritum	Cato_nig	Br	3.1	0.0	-3.1	0.0	-3.1	n/a	0.82	0.0	0.0	0.0	0.0	0.0	n/a	n/a
Cinclidium stygium	Cinc_sty	Br	1.6	4.7	**+3.1**	5.1	+3.5	-2.82	-2.48	0.0	0.0	0.0	0.0	0.0	n/a	n/a
Ctenidium molluscum	Cten_mol	Br	0.0	0.0	0.0	0.0	0.0	n/a	n/a	38.1	75.0	+36.9	44.8	+6.7	-1.45	-1.00
Fissidens adianthoides	Fiss_adi	Br	4.5	2.3	-2.2	1.7	**-2.8**	1.93	3.18	4.6	3.6	-1.0	2.4	-2.2	0.35	1.81
Fissidens osmundoides	Fiss_osm	Br	0.0	0.0	0.0	0.0	0.0	n/a	n/a	0.0	0.0	0.0	0.5	+0.5	n/a	-1.00
Leiocolea rutheana	Leio_rut	Br	4.8	3.3	-1.5	2.1	-2.7	1.70	1.81	3.3	1.1	-2.2	0.8	-2.5	1.72	2.10
Plagiomnium elatum	Plag_ela	Br	0.6	9.4	+8.8	2.1	+1.5	-0.92	-0.50	1.9	9.4	+7.5	0.0	-1.9	-0.55	1.20
Plagiomnium ellipticum	Plag_ell	Br	0.6	2.1	+1.5	2.1	+1.5	-1.0	-1.0	0.0	1.3	+1.3	0.0	0.0	-1.23	n/a
Rhizomnium pseudopunctatum	Rhiz_pse	Br	0.1	1.9	+1.8	3.5	+3.4	-1.19	-1.1	3.1	0.0	-3.1	0.0	-3.1	1.00	1.00
Scorpidium scorpioides	Scor_sco	Br	52.5	37.5	-15.0	0.0	-52.5	-1.0	0.64	44.4	35.9	-8.5	15.6	-28.8	1.48	1.22
Tomentypnum nitens	Tome_nit	Br	5.5	8.0	+2.5	10.0	+4.5	-0.89	0.24	0.0	0.0	0.0	0.0	0.0	n/a	n/a
Bartsia alpina	Bart_alp	Fo	0.9	1.2	+0.3	1.3	+0.4	-1.19	1.51	1.4	2.9	+1.5	0.6	-0.8	-1.20	3.18
Crepis paludosa	Crep_pal	Fo	1.1	4.8	+3.7	4.9	+3.8	-0.95	-0.32	3.1	1.0	-2.1	0.0	-3.1	0.99	1.00
Dactylorhiza incarnata ssp. *cruenta*	Dact_cru	Fo	0.9	0.5	-0.4	1.7	+0.8	1.90	-0.42	0.0	0.0	0.0	0.0	0.0	n/a	n/a

Species	abbreviation (see Fig. 3)	plant group	ci	cu	ci→cu	cm	ci→cm	*T* cu (df=16)	*T* cm (df=16)	oi	ou	oi→ou	om	oi→om	*T* ou (df=13)	*T* om (df=13)
Dactylorhiza incarnata ssp. *incarnata*	Dact_inc	Fo	0.0	0.0	0.0	0.0	0.0	n/a	n/a	0.4	0.0	-0.4	0.5	+0.1	1.49	1.22
Gymnadenia conopsea	Gymn_con	Fo	0.5	0.5	0.0	2.1	+1.6	1.61	1.01	3.1	0.3	-2.8	0.5	-2.6	0.99	0.82
Listera ovata	List_ova	Fo	0.0	1.5	+1.5	0.0	0.0	-1.63	n/a	1.1	0.9	-0.2	0.5	-0.6	1.78	2.61
Parnassia palustris	Parn_pal	Fo	0.8	0.5	-0.3	2.1	+1.3	1.39	-1.30	2.7	1.6	**-1.1**	1.9	**-0.8**	4.56	3.14
Pedicularis oederi	Pedi_oed	Fo	2.2	2.6	+0.4	2.2	0.0	-0.26	0.86	0.0	0.0	0.0	0.0	0.0	n/a	n/a
Saussurea alpina	Saus_alp	Fo	3.4	3.4	0.0	4.9	+1.5	0.26	0.85	2.2	2.3	+0.1	1.3	**-0.9**	-0.17	2.25
Saxifraga aizoides	Saxi_aiz	Fo	2.7	2.1	-0.6	1.3	-1.4	1.61	1.83	0.0	0.0	0.0	0.0	0.0	n/a	n/a
Thalictrum alpinum	Thal_alp	Fo	11.1	5.7	**-5.4**	16.9	+5.8	2.92	-1.96	7.4	6.9	-0.5	10.2	+2.8	0.49	-1.40
Tofieldia pusilla	Tofi_pus	Fo	2.1	1.6	-0.5	2.7	+0.6	2.42	-0.27	2.8	0.9	**-1.9**	1.3	**-1.5**	4.71	4.12
Triglochin palustris	Trig_pal	Fo	0.9	0.5	-0.4	0.5	-0.4	2.42	1.69	0.6	0.0	-0.6	0.5	-0.1	1.01	0.55
Carex atrofusca	Care_atr	Gr	6.9	0.5	-6.4	9.4	+2.5	1.00	-1.0	0.0	0.0	0.0	0.0	0.0	n/a	n/a
Carex buxbaumii	Care_bux	Gr	0.2	2.6	+2.4	9.4	+9.2	-0.93	-0.91	0.0	1.3	+1.3	0.0	0.0	-1.23	n/a
Carex capillaris	Care_cpl	Gr	2.8	2.1	**-0.7**	2.6	-0.2	2.93	2.36	3.4	1.9	**-1.5**	3.8	+0.4	2.99	1.82
Carex capitata	Care_cpt	Gr	2.8	7.0	+4.2	2.1	-0.7	-1.15	-1.19	0.0	0.0	0.0	0.0	0.0	n/a	n/a
Carex flava	Care_fla	Gr	2.0	3.5	+1.5	6.4	**+4.4**	-1.31	-2.82	7.2	4.8	-2.4	8.5	+1.3	0.92	-0.43
Carex hostiana	Care_hos	Gr	1.9	7.0	+5.1	5.4	+3.5	-0.50	-0.72	8.5	10.5	+2.0	10.7	+2.2	-1.00	-1.71
Carex microglochin	Care_mic	Gr	0.0	0.0	0.0	0.0	0.0	n/a,	n/a	0.0	0.5	+0.5	4.7	+4.7	-1.00	-1.00
Carex pulicaris	Care_pul	Gr	0.0	0.0	0.0	0.0	0.0	n/a	n/a	0.0	2.1	+2.1	2.1	+2.1	-1.00	-1.00
Eleocharis quinqueflora	Eleo_qui	Gr	6.1	1.3	-4.8	4.7	-1.4	1.39	1.18	0.0	0.0	0.0	2.1	+2.1	n/a	-1.00
Eriophorum latifolium	Erio_lat	Gr	6.7	7.9	+1.2	6.8	+0.1	-1.42	-1.66	6.5	11.6	+5.1	9.9	+3.4	-2.34	-2.06
Juncus castaneus	Junc_cas	Gr	0.3	0.0	-0.3	0.0	-0.3	n/a	1.02	0.0	0.0	0.0	0.0	0.0	n/a	n/a
Juncus triglumis	Junc_tri	Gr	1.1	1.3	+0.2	0.5	-0.6	1.93	1.06	0.0	0.0	0.0	0.0	0.0	n/a	n/a
Kobresia simpliciuscula	Kobr_sim	Gr	4.9	5.2	+0.3	4.0	-0.9	2.36	2.16	0.0	0.0	0.0	0.0	0.0	n/a	n/a
*Molinia caerulea**	Moli_cae	Gr	11.5	12.5	+1.0	4.1	**-7.4**	-0.82	4.61	10.1	22.3	**+12.2**	3.4	**-6.7**	-6.22	5.10
Schoenus ferrugineus	Scho_fer	Gr	0.0	0.0	0.0	0.0	0.0	n/a	n/a	8.8	4.7	-4.1	4.7	-4.1	1.00	1.00

*Betula nana**	Betu_nan	Sh	6.2	8.4	+2.2	0.6	-5.6	-0.71	1.51	0.0	0.0	0.0	0.0	0.0	n/a	n/a
*Betula pubescens**	Betu_pub	Sh	0.8	5.5	+4.7	4.9	+4.1	-2.25	-0.77	6.5	4.6	-1.9	1.5	-5.0	0.56	0.94
Salix myrsinifolia	Sali_myr	Sh	1.9	9.9	+8.0	0.5	-1.4	-1.03	0.70	3.1	6.8	+3.7	0.5	-2.6	-1.00	1.00
