# Supplementary material for: Can mowing restore boreal rich-fen vegetation in the face of climate change?
Source: PLoS One. 2019 Feb 19;14(2):e0211272. doi: 10.1371/journal.pone.0211272 (PMC6380559; doi:10.1371/journal.pone.0211272)
Supplement: S3 Table — The “Difference” column shows difference in mean value between the continental and oceanic sites for that variable. P values that show significant differences between sites (P<0.05) are shown in bold. (PDF) [file pone.0211272.s003.pdf]

	Continental (Sølendet)			Oceanic (Tågdalen)				
**Environmental variable**	Mean	Max	Min	Mean	Max	Min	Diff-erence	*P* value
Mean temperature sum (°C)	596	904	374	661	1029	397	65	**0.021**
Mean precipitation sum (mm)	641	862	471	1564	2254	968	923	**<0.001**
Peat depth (cm)	34	60	10	127	200	0	92	**0.001**
pH in water	7.1	7.5	6.0	6.6	7.1	5.9	-0.4	**0.005**
Electrical conductivity (µS)	120	195	64	163	303	52	43	0.145
Production (g/m^2)	153	312	76	119	265	41	-34	0.183
Median groundwater level (m)	-0.13	-0.06	-0.17	-0.05	-0.02	-0.11	0.08	**<0.001**
Maximum groundwater level (m)	0.44	0.76	0.22	0.08	0.19	0.03	-0.35	**<0.001**
Minimum groundwater level (m)	-0.42	-0.31	-0.56	-0.23	-0.10	-0.41	0.18	**<0.001**
